# The American Dental Association

**Published:** 1893-09

**Authors:** 


### The American Dental Association—1893.

Pursuant to adjournment the thirty-third annual meeting of the American Dental Association was held in Chicago, August 12th, 1893.

The meeting was held in Kindergarten College Hall. The President of the Association, Dr. J. D. Patterson, Kansas City, occupied the chair. After a brief opening address the following resolutions were offered by the Executive Committee:

WHEREAS, the date of our meeting, which was fixed for August 15th, under the expectation that it would immediately precede the opening of the World's Columbian Dental Congress, has been changed because of the change in the date of holding the Congress; therefore

1. *Resolved*, that the unanimous action of the executive committee in calling the meeting in advance of the day selected is hereby approved and declared to be legal and binding.

WHEREAS, it has been generally understood by the members that in order that more interest and work should be concentrated in the congress, the meeting of the association this year should be as nearly as possible of a merely formal character; therefore

2. *Resolved*, that the dues for the current year be remitted and the treasurer be instructed to give receipts in such form that a single payment shall cover the dues for the current and the coming year.

3. *Resolved*, that the meeting this year be adjourned without any election of officers, as under the constitution the effect of such non-election will be to make all officers elected last year hold over.

4. *Resolved*, that all records and transactions of this year be considered as merged in the proceedings for 1894 and so published, in order that in spirit and in name the officers elected last year shall not be considered to have held office and exercised their functions for two sessions.

5. *Resolved*, that the treasurer be instructed to pay all properly authenticated bills.

6. *Resolved*, that Old Point Comfort be selected as the place of meeting for next year.

The first and second resolutions were unanimously adopted without discussion. To the third resolution Dr. W. C. Barrett, Buffalo, N. Y., offered as an amendment, that a single ballot be cast for the re-election of all officers, in order to avoid establishing a precedent allowing officers to hold over. The amendment was not adopted; the original resolution being accepted.

By a unanimous vote Resolution V was adopted, and the treasurer instructed to pay all duly authorized bills.

Resolution VI elicited a spirited discussion.

Dr. J. Taft offered a resolution that other places than Old Point Comfort, especially those more central, be given a chance.

Dr. C. C. Carroll offered a vigorous protest against such informal "cut and dried" *resoluting*.

The claims of Lookout Mountain, San Francisco, Niagara Falls, Saratoga, were respectively urged.

Dr. L. D. Shepard, President of the Congress, stated that the Executive Committee in offering the aforesaid resolutions had acted in compliance with the duty laid upon them by the constitution of the association. It was within their province to accept or reject any or all of the resolutions. In conclusion he offered as a substitute for the sixth resolution that a nomination list be opened and the ballot taken. This was accepted, and as the result of the ballot it was announced that Saratoga had one vote, Lookout Mountain four, Old Point Comfort forty-five, Niagara Falls three, and San Francisco six. Old Point Comfort, Va., was accordingly declared the place of meeting for the next session.

On motion the reports of all other committees were deferred to next year, and the American Association adjourned to meet at Old Point Comfort in 1894.

